# Readability of information on colonoscopy preparation on the internet

**DOI:** 10.15171/hpp.2018.22

**Published:** 2018-04-18

**Authors:** Sarah A. MacLean, Corey H. Basch, Ashley Clark, Charles E. Basch

**Affiliations:** ^1^Icahn School of Medicine at Mount Sinai, 1 Gustave L. Levy Place, New York, NY 10029 USA; ^2^Department of Public Health, William Paterson University, 366 University Hall, Wayne, NJ 07470 USA; ^3^Health and Behavior Studies, Teachers College, Columbia University, 525 W. 120th Street, NY, NY 10027 USA

**Keywords:** Colonoscopy, Colon cancer, Readability, On-line information

## Abstract

**Background:** The incidence of colorectal cancer (CRC) has decreased in recent years, due in large part to increased screening, particularly through colonoscopy. This study aimed to examine the level of readability of information on colonoscopy preparation written on 100 websites that were found via an internet search.

**Methods:** In this cross-sectional study, the content of the first 100 websites in English found via an internet search were analyzed using established readability scales. Websites were compared based on whether they had a commercial or non-commercial URL extension.

**Results:** The majority of websites were found to have information of a difficult reading level. Less than 10% of websites had an easy reading level. Readability did not differ significantly based on URL extension.

**Conclusion:** The information currently posted on the internet regarding preparation for colonoscopy is written at a difficult reading level. If information presented was both accurate and easier to read, it could benefit a greater proportion of the general public and help inform decisions about preparing for a colonoscopy.

## Introduction

The incidence of colorectal cancer (CRC) has decreased in recent years, due in large part to increased screening, particularly through colonoscopy.^1^ A recent report from the Centers for Disease Control and Prevention indicated that, in the United States, during the time span ranging from 2002 to 2010 there was an increase in CRC screening from 52.3% to 65.4% in 50 to 75-year-olds. A meta-analysis with a pooled analysis of nearly 1.5 million individuals revealed that colonoscopy was associated with a 61% relative risk reduction in the incidence of CRC.^2^ The benefit of colonoscopy is highlighted when it is received for screening versus diagnostic purposes.^2^

Despite these advances in CRC screening, over one in three eligible patients (34.6%) do not undergo screening.^1^ A successful colonoscopy requires “preparation” by the patient to clear the bowel and allow adequate visualization to detect all lesions greater than 5 mm.^3^ In a prospective study at 18 medical centers, inadequate preparation was found in one third of colonoscopies.^4^ This is noteworthy because the detection rate of polyps or suspicious masses is significantly higher in patients who had adequate preparation compared to patients with inadequate preparation.^4^ In one study, almost half of the patients reported at least mild discomfort with the bowel preparation process.^4^ In a national survey of gastroenterologists’ opinions on factors associated with suboptimal bowel preparation, 29% reported patients’ not understanding bowel preparation instructions and 21% reported patients’ low level of education.^5^

Research indicates that people frequently search for information related to colonoscopy preparation on the internet.^6^ Information posted online can be misleading. In particular, any person posting on the internet can claim expertise in a subject and pages can be “official looking” while not being posted by a reputable source.^7^ Individuals may find it difficult to evaluate the veracity of information found on the internet, making them vulnerable to misunderstanding.^7^ Further, information that is credible may be difficult to understand. It has been suggested that information be written at a sixth grade reading level, as this could increase the likelihood that it will be understood by the general public.^8^ We did not identify any published studies on the readability of information about preparation colonoscopy found on the internet. This study, was, therefore, conducted to examine the level of readability of information on colonoscopy preparation written on 100 websites that were found via an internet search.

## Materials and Methods

The methods were adapted from other studies of readability.^9,10^ To create the sample of websites, the internet browser was cleared and the keywords “colonoscopy preparation” were entered to conduct the search. The URLs of the first 100 English language websites were included in the sample.

Readable.io is a service recommended by Medline, which generates readability scores using several commonly-recommended readability tests.^11^ Five readability tests we used included: Flesch-Kincaid Grade Level (FKGL), Gunning Fog Index (GFI), Coleman-Liau Index (CLI), Simple Measure of Gobbledygook (SMOG) Grade Level, and Flesch-Kincaid Reading Ease (FRE). The scores on these scales can be grouped to designate a website as “easy” (grade <6), “average” (grade 6-10), or difficult (grade >10) in terms of readability.

Websites were classified based on their URL extension into group 1 (.org, .gov, or .edu) and group 2 (.com, .net. or other). Descriptive statistics were calculated and statistical tests were performed using SPSS (v23). Categorical variables were tested using the chi-square test of association and independent sample *t* tests were used to test continuous variables. When an expected cell count was less than 5, Fisher’s exact tests were used in place of chi-square tests. Results were considered significant if *P* < 0.05.

## Results

Less than 10% of websites were scored as easy (grade <6), which is the goal of Information intended for the general public (Table 1).^8^ Even by increasing the goal grade level to Grade 10, which would thus include websites rated as easy and average, only one test (FKGL) found that the majority of websites would have acceptable readability. Two tests (SMOG and FRE) found that less than 30% of websites would meet this less stringent criterion. Four of the five tests determined that the majority of the websites were rated as difficult (grade >10). Based on two of the tests (SMOG and FRE), at least 72% of websites had difficult readability.

There was little difference found between group 1 (.org, .gov, or .edu) and group 2 (.com, .net, other). Based on independent sample *t* tests, the mean readability scores of sites did not differ significantly between the 2 groups. Based on one test (FKGL), compared with group 1, a greater proportion of group 2 websites appeared to be of average or difficult readability (*P*=0.042). Overall, these results thus show that the content posted on less than 10% of these sites was not “easy” to read.

## Discussion

To our knowledge, this is the first study to assess the readability of materials related to colonoscopy preparation on the internet. Our results demonstrate that, despite the recommendation that information be presented to the general public at a sixth grade reading level,^8^ most of the websites addressing colonoscopy preparation presented content that was at a greater than 10th grade reading level based on four of the 5 tests conducted. These findings are consistent with findings from other studies that suggest reading materials on the internet may be difficult for many individuals to interpret.^9,10^ Future studies could specifically examine the credibility of information on colonoscopy preparation presented on the internet, and discover ways to improve the readability of material presented. For instance, the National Institutes of Health has suggested that visual presentation and representation be carefully considered in constructing materials, as it plays a part in the effectiveness of conveying information.^11^

In addition, the creation of readable print materials conveying instructions through video presentation, such as YouTube, is a promising strategy for reaching the large proportion of Americans with low levels of reading literacy. For such videos to help consumers make informed decisions about colonoscopy preparation, they must be designed in ways that are acceptable and will attract viewers. A study of colonoscopy preparation on YouTube revealed that both consumer and healthcare generated video content were popular, although content created by healthcare professionals was viewed more often.^12^ Additional research is needed to conceptualize and design such videos in ways that not only communicate accurate information, but are culturally acceptable and attract views by the intended audience. Identifying the best practices for pairing video content with easy to read text would be an asset in this field of study.

Health literacy has been shown to be an important factor in understanding colonoscopy preparation procedures.^13,14^ Since successful preparation prior to the procedure can influence adenoma detection rate,^15^ it is crucial that patients understand the preparation regimen. Researchers report a significant cost associated with poor quality bowel preparation.^16^ Rates of inadequate bowel preparation remain high,^17,18^ however, and are associated with missed polyps.^17,19^

The limitations of this study include the cross-sectional design, and the exclusive use of readability tests versus other tests that offer further insight into the understandability of materials. Based on the results of this preliminary study, one cannot conclude that the posting of more easily readable information about colonoscopy preparation would lead to more successful preparation. Nevertheless, our results suggest that if information about colonoscopy preparation were presented with a lower level of readability, the information may be accessible to a larger proportion of the public. Furthermore, if more was understood about what is involved in colonoscopies, adherence to guidelines could increase.

Readability is one metric to consider, but it does not guarantee understanding. Even once instructions are understood, efforts are needed to improve the extent to which they are followed. Additional research is needed to improve understanding about ways to communicate with the public in ways that increase the likelihood of adequate colonoscopy preparation.

## Ethical approval

The IRB at William Paterson University does not review studies that do not involve human subjects and automatically consider them to be exempt, and the study was found to be exempt by the IRB at Teachers College, Columbia University.

## Competing interests

The authors declare that they have no competing interests.

## Funding

This research was unfunded.

## Authors’ contributions

SAM analyzed the data and drafted the manuscript. CHB conceived the project and assisted with drafting the manuscript. AC collected the data. CEB assisted with data analysis and drafting the manuscript. All authors approved of the final manuscript.

**Table 1:** Descriptive statistics on the readability of all sites (N = 100) and comparison of websites based on URL type

**Test**	**Min.**	**Max.**	**Mean**	**SD**	**N**			**Mean**	**SD**	**Group 1 ** ^b^	**Group 2 ** ^c^	***P*** ^d^	**Group 1**			**Group 2**			***P *** ^e^
					**Easy**: Grade <6	**Avg**: Grade 6-10	**Diff**: Grade >10						**Easy**	**Avg.**	**Diff.**	**Easy**	**Avg.**	**Diff.**	
FKGL	2.3	15.2	9.6	2.6	9	49	42	9.6	2.6	10.0	9.3	0.200	5	14	22	4	35	20	**0.042**
GFI	1.2	18.3	10.4	3.6	9	35	56	10.4	3.6	10.7	10.2	0.527	3	12	26	6	23	30	0.512
CLI	4.4	18.0	10.8	2.5	2	36	62	10.8	2.5	11.3	10.4	0.097	1	12	28	1	24	34	0.489
SMOG	6.0	16.4	11.5	2.1	0	27	73	11.5	2.1	11.8	11.2	0.157	0	10	31	0	17	42	0.655 ^f^
FRE ^a^	9.3	87.6	49.5	16.2	2	26	72	49.5	16.2	47.1	51.2	0.211	1	9	31	1	17	41	0.753

^a^ Scoring is as follows: Easy: score 80-100; average: score 60-79; difficult: score 0-59. ^b^Group 1: .org, .gov, .edu (n = 41). ^c^Group 2: .com, .net, other (n = 59). ^d^ Independent sample *t* test. ^e^ Fisher’s exact test. ^f^Chi-square test.
